# African Swine Fever in Mongolia: Course of the Epidemic and Applied Control Measures

**DOI:** 10.3390/vetsci7010024

**Published:** 2020-02-17

**Authors:** Martin Heilmann, Amarsanaa Lkhagvasuren, Tuvshinbayar Adyasuren, Bodisaikhan Khishgee, Bayartungalag Bold, Ulaankhuu Ankhanbaatar, Guo Fusheng, Eran Raizman, Klaas Dietze

**Affiliations:** 1 Food and Agriculture Organization of the United Nations, Ulaanbaatar 14201, Mongolia; amar_lh@yahoo.com; 2 General Authority for Veterinary Services, Ulaanbaatar 210349, Mongolia; tuvshee1116@gmail.com (T.A.); bodisaikhan@dvab.gov.mn (B.K.); bayartungalag@dvab.gov.mn (B.B.); 3 State Central Veterinary Laboratory, Ulaanbaatar 17024, Mongolia; ulaankhuu@scvl.gov.mn; 4 Food and Agriculture Organization of the United Nations, Bangkok 10200, Thailand; Fusheng.Guo@fao.org; 5 Food and Agriculture Organization of the United Nations, 1063 Budapest, Hungary; Eran.Raizman@fao.org; 6 Friedrich-Loeffler-Institut, 17493 Greifswald, Germany; Klaas.Dietze@fli.de

**Keywords:** epidemiology, backyard pig, swill feed, disease control, outbreak investigation

## Abstract

African swine fever (ASF) is spreading rapidly in Asia and was confirmed in Mongolia on 10 January 2019. Following the outbreak confirmation, a state emergency committee was established with representation from municipal authorities and other relevant authorities including the General Authority for Veterinary Services, National Emergency Management Agency, General Agency for Specialized Inspection, and the Ministry of Environment and Tourism. The committee provided recommendations and coordinated closely with the State Central Veterinary Laboratory to ensure quick outbreak investigation and response. In addition to outbreak investigations, sampling took place at farms and food premises and suggests a link between the outbreaks and swill feeding practices among backyard pig farmers. Upon government request, the Food and Agriculture Organization of the United Nations (FAO) deployed an expert team to assist in identifying risk factors for the disease spread and provide recommendations as how to improve disease prevention and response. Following the control measures from the involved agencies, the epidemic was successfully controlled and declared over on 11 April 2019. In total, the epidemic affected 83 pig farming households and led to a total of 2862 dead or culled pigs in eleven districts of seven provinces in Mongolia.

## 1. Background

African swine fever (ASF) is a viral disease that recently spread to East and Southeast Asia [1]. The virus affects domestic and wild pigs, leading to various symptoms from chronic or persistent infection to acute hemorrhagic fever with high mortality [2]. However, the disease poses not only a threat to pigs’ health, but also to food security and the livelihoods of stakeholders along the entire pig value chain. This is all the more important for the Asian region, which accounts for over half of the global pig meat production [3]. The ASF epidemic in Asia has already demonstrated a significant impact on global markets including rapidly rising prices for pig meat in 2019 [4]. Since December 2007, ASF was reported in the Russian Federation with subsequent outbreaks between 2008 and 2009 in both domestic pigs and wild boars in the southern regions of the Russian Federation [5]. In August 2018, the disease was reported in China and was spreading rapidly.

**Figure 1:**
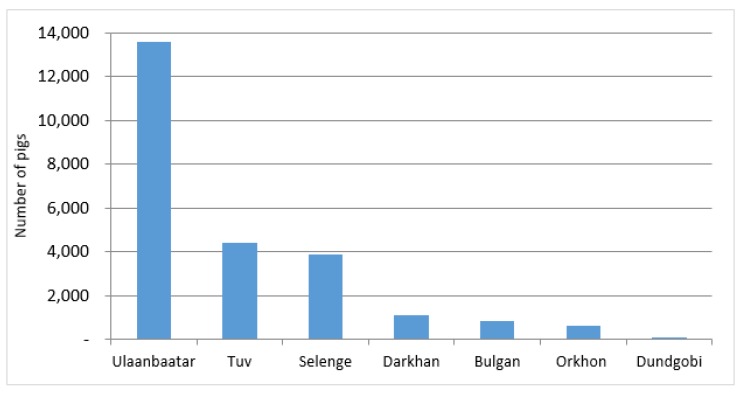
Number of domestic pigs in affected provinces as per 2018.

Mongolia consists of 21 provinces (*aimags*) subdivided in a total of 331 districts (*soums*). At the end of 2018, Mongolia counted 27,819 domestic pigs [6]. Approximately 90 percent of the domestic pig population is located in the Bulgan, Orkhon, Selenge, and Darkhan Provinces and Ulaanbaatar City (Figure 1). Pigs are dominantly kept in semi-intensive backyard production systems as defined by the Food and Agriculture Organization of the United Nations (FAO) [7]. In a Mongolian context, this means more specifically that pigs are confined in very simple pens located within the perimeter area of the producer’s house and pig farming is run mainly by household members.

Early detection of animal diseases is predominantly building on passive reporting. Data collection for animal disease surveillance is not conducted systematically for both the domestic pig and wild boar population. The latter is recognized as a potential reservoir for ASF [8]. The country has suffered several incursions and outbreaks of transboundary animal diseases over the past years including classical swine fever, foot and mouth disease, sheep pox and peste des petit ruminants.

In early 2019, the disease was confirmed in Mongolia. This paper aims to give insights into the course of the epidemic and the applied control measures in the Mongolian context.

## 2. The Course of the ASF Epidemic

On 10 January 2019, the State Central Veterinary Laboratory (SCVL) confirmed the first ASF outbreak in Mongolia in the Bulgan district, in Bulgan Province. The index herd was located near the capital of Bulgan Province and had a herd size of 38 pigs. Outbreak investigations revealed that the animals were free roaming with access to a local garbage dump that was regularly frequented by other animals. Contact to wild pigs was not reported. Pigs in the index herd were additionally fed with swill feed from food premises located near the capital. The pig farmer contacted the local veterinarian initially due to sudden death in animals and sick animals showing high fever and red ears.

The initial samples from the index herd included ten tissue samples from dead pigs (kidney, muscle, spleen, and lymph nodes) were collected in Bulgan Province and laboratory diagnosis was performed at the SCVL using reverse transcription polymerase chain reaction (RT-PCR), quantitative RT-PCR, and antibody enzyme-linked immunosorbent assay (Ab-ELISA) techniques. On 16 January, the samples were sent to and subsequently confirmed for ASF by the Pirbright Institute, World Organization for Animal Health (OIE) ASF reference laboratory. On 15 January, following confirmation by the SCVL, the Government of Mongolia (GoM) notified the OIE about the outbreak.

**Figure 2:**
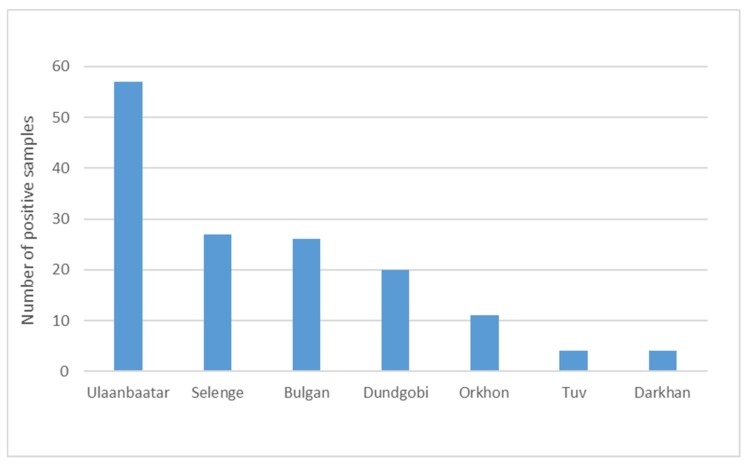
Total number of African swine fever positive samples by province obtained between 8 January and 9 February 2019 in Mongolia.

**Figure 3:**
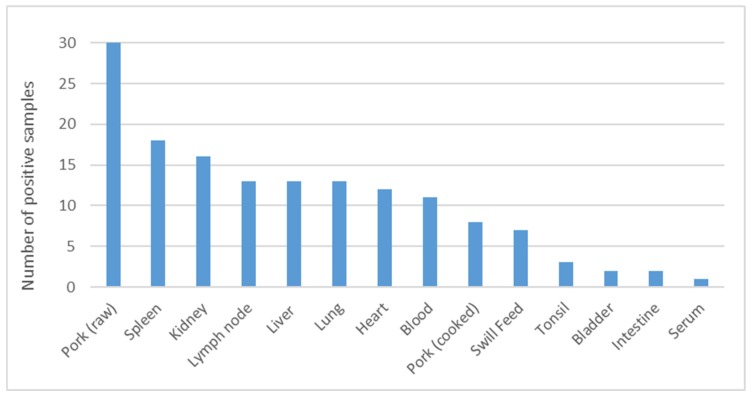
Total number of African swine fever positive samples by category obtained between 8 January and 9 February 2019 in Mongolia.

**Figure 4:**
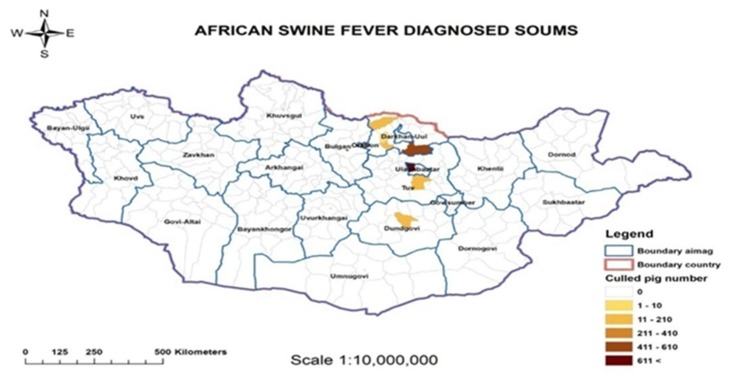
African swine fever outbreaks in Mongolia (2019) per district (*soum*) with the total number of culled pigs.

Overall, the sampling period took place between 9 January and 8 February, 2019 and included pig farms as well as food premises. Out of a total of 279 samples, 149 tested positive for ASF. The total number of ASF positive samples is shown by province (Figure 2) and category (Figure 3). All samples from fat (n = 4) were negative and are thus not listed. The category pork is divided in raw and processed (cooked) pork. The veterinary service at the central level guided the local authorities on how to conduct surveillance targeting pig farmers in all provinces of Mongolia. The rollout was supported by public and private veterinarians who conducted active surveillance including daily phone interviews in affected areas to find cases. Outbreak investigations in affected farms followed a structured questionnaire that included the herders’ personal information, farm coordinates, animal movement, and feed source as well as any unusual signs in animals over the past few weeks. Further outbreaks were subsequently confirmed in different provinces including Orkhon, Darkhan, Tuv, Dundgobi, and Selenge. In total, the epidemic affected 83 pig farming households and led to a total of 2862 dead or culled pigs in eleven districts of seven provinces including Ulaanbaatar City, as shown in Table 1 and illustrated in Figure 4, respectively.

## 3. Control Measures

To facilitate coordination, the GoM established a state emergency committee following the initial outbreak confirmation on 15 January 2019. The committee was established with representation from municipal authorities as well as other relevant authorities including the General Authority for Veterinary Services (GAVS), National Emergency Management Agency (NEMA), General Agency for Specialized Inspection (GASI), and the Ministry of Environment and Tourism. The committee provided recommendations for coordinated outbreak response and worked closely with the SCVL to ensure quick outbreak investigation and response in the field. The applied control measures described here drew largely upon the recommendations from the committee. These included to support the outbreak management by stopping free-roaming pigs and any other animal movement such as for breeding in affected outbreak areas. Recommendations further included not to feed swill to pigs including specifically not using food waste from food premises. The recommendations were still upheld after the outbreak, although not made obligatory through legally binding law. The veterinary services also provided guidelines to the local authorities and farmers with information about ASF prevention and its control. Additionally, veterinary services raised awareness among the public through television and brochures about the risks, particularly on swill feeding, free-roaming pigs, and low biosecurity levels. The GAVS facilitated reporting by setting up a hotline, which was used intensively by local veterinary services in case they observed visibly sick or dead pigs. As part of the control efforts, wildlife authorities were contacted to ensure and confirm that no increased wild boar mortality was reported in order to rule out any potential spill-over. Unlike many of the countries infected, no cases of ASF have been reported in wild boars in Mongolia.

Agencies in charge of veterinary and inspection services applied movement control and quarantine measures. Quarantine included strict movement control of people as well as animals and was applied within 24 h in all outbreak areas following laboratory confirmation. The quarantine duration varied among the different outbreaks areas, with a minimum of 14 and a maximum of 67 days, respectively, with a median of 25 days. After the quarantine, restrictions continued and were gradually lifted, allowing for limited movement of people but still no animal production. On average, any remaining restrictions were canceled after 39 days following the outbreak confirmation. Table 1 provides more information about the ASF outbreaks at farm level by province and district including control measures and impacts on the pig population. Additionally, pig products, raw, half, or fully processed, were immediately banned from import upon outbreak confirmation including any pork used during food manufacturing processes. The general import ban was canceled after two weeks on 4 February, 2019 by order of the Ministry of Food, Agriculture, and Light Industries. Another integral part of the biosecurity measures included the cleaning and disinfection of farms, food premises, and related production facilities as well as transport vehicles using the same product as above-mentioned. Involved personnel were equipped with personal protective equipment, which was provided by the veterinary services and authorities at the provincial level.

In parallel to the confirmed outbreaks, the veterinary service at the central level sent out official letters to the local authorities on behalf of the chief veterinary officer to ensure the coordinated implementation of control measures including on culling by shooting, disposal, cleaning and disinfection, and movement control. The culling and disposal procedures were largely based on an existing guideline on ‘Disposal of animal carcasses’ that was approved in October 2018 by government order A-52. A total of 2862 domestic pigs died or were culled, which corresponded to ca. ten percent loss of the total pig population as per pig numbers from 2018 [6]. The dead animals were disposed of by burial in at least two meter deep holes outside residential areas. They were then burned with the help of highly flammable materials such as petrol and tires. Carcasses burned fully or sometimes partly only to ashes given the climatic conditions at the time of the outbreak, which were characterized by an average temperature of −18 degrees Celsius in the affected provinces in January 2019 [6]. Following the burning, a broad-spectrum virucidal disinfectant (brand name: Hi-Cop) was poured over the carcasses before refilling the hole with soil. At the end, a sign was installed to indicate a burial point and a fence was put around the disposal area. Pig products were disposed similarly, although often burned at common garbage sites. Pig farmers were not compensated for the losses during the outbreak.

On 24 January, the GAVS requested the FAO to organize an emergency mission to undertake a rapid assessment and advise on how to improve the outbreak response. Following the official request, the Emergency Management Centre for Animal Health at FAO (EMC-AH) organized a rapid deployment team in collaboration with OIE. The mission took place from 18 to 22 February 2019 and supported GAVS in identifying risk factors for the spread of disease, gaps in the outbreak response along with recommendations to improve disease prevention, surveillance and response.

Concluding with all official control measures, the outbreak was declared over on 11 April 2019.

## 4. Discussion

Despite the challenging environment with limited resources for veterinary services and a limited attention given to pig production and pig disease as compared to the dominant ruminant and equine species, the Mongolian authorities comprehensively addressed the ASF incursion in 2019. This description of the control measures allowed us to draw some conclusions and lessons relevant for countries operating in comparable settings.

The outbreak investigation suggest a link between the outbreaks and swill feed practices in backyard pig farms. The swill feeding practice, or more generally feeding table scraps or uncooked organic refuse in which the virus can persist and remain infectious if it is not previously cooked, is a commonly observed practice among backyard producers in the region [4]. In fact, government officials observed some backyard pig farmers who commonly purchased pig feed from wet markets or even brought large containers of 10–20 liters to food premises to ask them to fill them with food waste against a small remuneration. Food premises, on the other hand, often use imported pig products given that the domestic production is small compared to the demand, and domestic pig products are usually only consumed locally. Even though it was recommended to avoid swill feeding, respecting the socio-economic realities and common practices, it was not officially banned. The respective sensitization among the pig farming households at least provided the farmers with the required information to take risk-based decisions. The detection of the ASF virus genome in food waste commonly used for swill feeding in Mongolia highlights the possibility of swill being a likely entry point for ASF into pig production units. The need to properly heat treat swill before feeding it remains of paramount importance in the case that the use of swill as feed cannot be avoided. As the ASFV genome will remain detectable in properly heated swill that would no longer contain the infectious virus, it can still serve as an indicator for the potential risk of exposure through swill feeding.

As per information from GAVS, where companies have to acquire permission and indicate import quantities, pig products from China dominate the market and are preferred among larger companies given their relatively low price and cheap transport via the trans-Mongolian railway.

A clear action has been taken with the ban on imported pig products following the outbreak confirmation. However, the ban did not take into consideration the ASF disease status of the country of origin (it also included countries free of ASF), which caused adverse and unnecessary impacts on the pig sector and probably opened the door to smuggling. In the absence of a thorough risk assessment, the general import ban was lifted two weeks later and allowed ASF positive countries such as China to export products to Mongolia again. Following the outbreak experience, the government has put the veterinary services fully in charge of undertaking risk assessments of companies importing pig products to ensure risk-based decision-making in the future, and to avoid overlapping responsibilities with other authorities.

A major challenge during the outbreak was the communication and coordination with the various agencies involved in the response to the outbreak including horizontally across agencies as well as vertically from the central to local level. Despite the existing capacity and knowledge, the outbreak investigation process was incomplete in some outbreak locations. While quarantine measures were applied in a timely manner and strictly enforced due to centralized and defined chains of command, the resulting impacts on livelihoods should be carefully considered. Under the current policy, the people at any premise under quarantine were not allowed to leave the premise at all. This implied, for example, that adults were not able to go out for daily work and children could not attend school. Veterinary services applied such strict measures on movement control to minimize the risk of exposure in light of climatic conditions that made the application of basic biosecurity measures more challenging and sometimes impossible. The overall compliance with this measure was high, with only a few exceptions that did not follow. Among the key issues in the early stages was that veterinary services sometimes had no access to premises to conduct outbreak investigation due to unclear and overlapping areas of authority with other agencies in charge of emergency management and specialized inspection. Furthermore, despite an existing guideline, the selection of affected pigs to be culled and the disposal procedures sometimes varied, depending on the provincial authorities. Furthermore, the culling of animals and their disposal in infected premises was not subject to animal welfare or environmental regulations and was handled by a private entity without supervision from relevant authorities. Additionally, the veterinary services enforced a two year restocking ban policy while the OIE Terrestrial Animal Health Code only suggests 40 days. Instead of this rather long latency that may prevent pig farmers from restarting their businesses, compliance with minimum biosecurity standards may be more pragmatic. Veterinary officials argue, however, that many of the current backyard facilities are hard or impossible to clean or disinfect, especially due to climatic conditions, and thus require a prolonged restocking period. Some research indeed suggests that the virus’s stability in feces depends largely on the temperature and can extend beyond the suggested duration [9,10].

The cleaning and disinfection posed particular challenges given that the average temperature was far below 0 °C during the outbreak period. The effectiveness remains questionable under the Mongolian conditions and may have caused incomplete disinfection. Further disinfection may need to be considered once temperatures rise above the freezing mark.

Additionally, pig farmers did not receive any compensation for the losses during the outbreak, which may have adverse economic effects on people that often have a challenging socio-economic background. A compensation policy can help to facilitate future cooperation for timely disease reporting and outbreak management and to avoid the spread of disease through illegal slaughtering or smuggling. Following the outbreak experience, the GAVS is currently encouraging the reporting of sick animals within twelve hours as per Article 15 of the Animal Health Law. Implementation and communication from the central to local level takes time, however, given the mere size of the country, sparse population, and institutional setup. Furthermore, the government is currently in contact with OIE to adopt, translate, and disseminate information about ASF to pig farmers in order to improve reporting and early detection. Although cases of ASF in wild boars have not been reported in Mongolia, the government is planning to conduct a study this year in collaboration with the School of Veterinary Medicine in Ulaanbaatar to investigate the situation.

Clear and explicit internal standard operating procedures can help in the future to improve the process and ensure the effective control measures nationwide. Such procedures should address both backyard as well as medium to large-scale pig productions. They should further be developed jointly with private sector representatives and be specific for ASF. Similar, however not identical, standard operating procedures should be developed for other transboundary animal diseases (e.g., foot and mouth disease, avian influenza, peste des petit ruminants, and sheep and goat pox).

## 5. Conclusions

Despite operating in a challenging environment, authorities in Mongolia successfully controlled the ASF epidemic after the initial incursion thanks to quick action and strictly applied control measures. The predominant backyard production of pigs and large distances between holdings in often sparsely populated areas played an important role in this outbreak scenario. Lessons have been taken to control and prevent similar outbreaks in the future specifically including the establishment of (i) regular information sharing among pig holders; (ii) risk assessments for imported pig products streamlined and enforced by the veterinary services; and (iii) an annual nationwide surveillance for pig diseases in domestic and wild pig populations on ASF and other diseases.

## Figures and Tables

**Table 1:** African swine fever cases in Mongolia (2019) by province and district including control measures and impacts on pig population.

Province/City	District	First Clinical Signs	Confirmed Diagnosis	Number of Pig Holders	Sick or Dead Pigs Prior to Confirmation	Culled Pigs Following Confirmation	Total Pig Loss	Quarantine Start	Quarantine End	Cancellation of Restrictions
Bulgan	Bulgan	4-Jan-19	10-Jan-19	12	112	335	447	10-Jan-19	4-Feb-19	18-Feb-19
Orkhon	Jargalant	10-Jan-19	13-Jan-19	2	115	130	245	14-Jan-19	28-Jan-19	11-Feb-19
Bayan-undur	10-Jan-19	13-Jan-19	8	151	42	193	14-Jan-19	28-Jan-19	11-Feb-19
Tuv	Sergelen	3-Jan-19	11-Jan-19	1	17	13	30	12-Jan-19	3-Feb-19	9-Feb-19
Dundgobi	Saintsagaan	12-Jan-19	13-Jan-19	1	9	37	46	14-Jan-19	28-Jan-19	11-Feb-19
Darkhan	Darkhan	10-Jan-19	15-Jan-19	3	24	49	73	16-Jan-19	30-Jan-19	10-Feb-19
Selenge	Orkhon	14-Jan-19	15-Jan-19	4	12	66	78	15-Jan-19	11-Feb-19	3-Mar-19
	Mandal	16-Jan-19	19-Jan-19	23	286	318	604	19-Jan-19	27-Mar-19	11-Apr-19
	Sant	23-Jan-19	24-Jan-19	1	1	4	5	24-Jan-19	12-Feb-19	4-Mar-19
	Tsagaannuur	19-Jan-19	24-Jan-19	4	5	27	32	24-Jan-19	4-Mar-19	12-Mar-19
Ulaanbaatar	Songinokhairkhan	6-Feb-19	8-Feb-19	24	435	674	1109	8-Feb-19	24-Feb-19	10-Mar-19
Total				83	1167	1695	2862			
